# Total Leucocyte Count, C-reactive Protein and Neutrophil Count: Diagnostic Aid in Acute Appendicitis

**DOI:** 10.4103/1319-3767.48969

**Published:** 2009-04

**Authors:** Sheikh Muzamil Shafi, Misbha Afsheen, Farooq A. Reshi

**Affiliations:** Postgraduate Department of Surgery, Government Medical College, Srinagar, India; 1 Postgraduate Department of Surgery, SMHS Hospital, Srinagar, India

**Keywords:** Acute appendicitis, C-reactive protein, neutrophil count, TLC

## Abstract

**Background/Aim:**

Acute appendicitis is one of the most common acute intraabdominal affections seen in surgical departments, which can be treated easily if an accurate diagnosis is made in time. Otherwise, delay in diagnosis and treatment can lead to diffuse peritonitis.

**Materials and Methods:**

A study was conducted on 110 patients who were operated for acute appendicitis to determine the role and predictive value of the total leucocyte count (TLC), C-reactive protein (CRP) and percentage of neutrophil count in the diagnosis of acute appendicitis. Preoperative TLC, CRP and percentage of neutrophil count were determined and were compared with the results of the histopathology of the removed appendix.

**Results:**

Of all the patients studied, 92 had histopathologically positive appendicitis. The TLC was found to be significantly high in 90 patients who proved to have acute appendicitis, whereas CRP was high in only 88 patients and neutrophil percentage was raised in 91; four had a normal CRP level. Thus, TLC had a sensitivity, specificity and positive predictive value of 97.82%, 55.55% and 91.8%, respectively. CRP had a sensitivity, specificity and positive predictive value of 95.6%, 77.77% and 95.6% respectively. Percentage of neutrophil count had a sensitivity, specificity and positive predictive value of 98.9%, 38.88% and 89.21%, respectively. When used in combination, there was a marked improvement in the specificity and the positive predictive value to 88.04% and 98.7%, respectively.

**Conclusion:**

The inflammatory markers, i.e., TLC, CRP and neutrophil count can be helpful in the diagnosis when measured together as this increases their specificity and positive predictive value.

General abdominal surgeons have been facing acute appendicitis from hundreds of years. Its accurate preoperative diagnosis still remains an impasse. Its accurate preoperative diagnosis still remains elusive. The overall negative laparotomy rate remains at about 20%.[1] In women of a childbearing age, this rate is nearly doubled because of the prevalence of gynecologic diseases, the figure being as high as 30–50%.[2] Among young male patients, the negative appendectomy rate is relatively low (5–22%).[3] In young children, the diagnosis may be incorrect in 30–46% of the cases.[2,4]

Despite the refined investigations there is no solution for the diagnostic dilemma of acute appendicitis: no particular test can reduce the rate of negative appendicectomy to zero. Based on unaided clinical diagnosis, the usually accepted figure for negative appendicectomy of about 15–30% is no longer acceptable. This figure can and ought to be reduced by supplementary measures.[1]

It has been well documented that there are certain acute-phase reaction proteins, including C-reactive protein (CRP), which are raised in various inflammatory conditions. If CRP can be added to the already existing laboratory tests, the diagnosis of acute appendicitis with clinically suggestive signs can be made with a fair degree of accuracy and, as such, unnecessary appendectomies can be avoided.

The purpose of this study was to study the preoperative leucocyte count, percentage of neutrophil count and CRP levels (triple test) in patients suspected of having acute appendicitis and to evaluate the preoperative diagnostic assurance and the predictive value of these tests in patients with acute appendicitis who underwent appendectomy at a later stage.

All the three tests (triple test) are easily-available blood tests, are not very expensive and the definite advantage is that they can be obtained within about 1-2 h. Thus, the surgeon on call can decide about the management of patients suspected of acute appendicitis well in time before complications ensue.

## MATERIALS AND METHODS

The study was conducted on patients with a clinical diagnosis of acute appendicitis, who underwent appendectomy at a later stage. These data were collected between May 2007 and April 2008. A total of 110 patients who were included in the study were operated for acute appendicitis at the Department of General Surgery, Govt. Medical College, Srinagar. Only those patients who presented within 12 h of onset of symptoms were included in the study. Interval appendicectomy were excluded from the study. Decision to operate was not influenced by the preoperative levels of these tests.

All the patients were operated for appendicitis on the basis of history, physical findings and relevant clinical data. Postoperatively, the removed appendix was sent for histopathological examination. Based on histological features of the removed appendix, the patients were divided into three groups as follows:

Group A: Normal appendix.

Group B: Inflamed appendix (simple appendicitis).

Group C: Perforated/gangrenous appendix (complicated appendicitis).

A review of their preoperative total leucocyte count (TLC) and CRP levels and percentage of neutrophil count was made. The sensitivity, specificity and positive predictive value of these tests were calculated.

The cut-off value for white cell count was taken as 11 × 10^6^/L. This value was selected arbitrarily as it corresponds to the elevated TLC. In our set up, the rapid latex agglutination slide test was the standard for the qualitative and semiquantitative ***in vitro*** determination of CRP in the sample. For semiquantitative determination, serum dilutions were prepared with the diluent provided with the commercially available CRP kit according to the following table:

**Dilution****C-reactive protein (mg/L in the undiluted sample)**1 + 1121 + 2181 + 3241 + 4301 + 536Etc.

Each dilution was tested according to the qualitative procedure described above until no further agglutination was observed. The CRP concentration was then estimated from the last dilution with visible agglutination.

CRP (mg/L) = Highest dilution with a positive reaction × reagent sensitivity (6 mg/L)

Percentage of neutrophil was considered elevated when >75%.

The number of patients with (1) both values normal, (2) only leucocyte count raised, (3) only CRP level raised and (4) both values raised were calculated in each of the three groups.

## RESULTS

A total of 110 patients were included in this study of whom 18 had a normal appendix histopathologically (Group A), giving an overall negative appendicectomy rate of 16.36%. In this study, 74 (67%) patients were males and 36 (33%) were females, the male to female ratio being 2:1. The age range was 8–69 years, with a median age of 20.3 years.

Among the 92 patients who had appendicitis, 79 had an inflamed appendix (Group B, simple appendicitis) and 13 had a ruptured/perforated/gangrenous appendix (Group C, complicated appendicitis).

The TLC was elevated in 90 patients and CRP was elevated in 88 cases among the patients with positive histopathology (Group B + C). Four patients had normal CRP and two patients had normal TLC. Of the 18 patients with negative appendix, 14 patients had a normal CRP level and only 10 patients had normal TLC. Again, in patients of Groups B and C, 77 had both TLC and CRP value raised and 15 patients had one or both values in the normal range. Of the 18 patients with negative appendix, two patients had both TLC and CRP values raised and the rest of the 16 patients had one or both values in the normal range.

When all the three parameters were combined (TLC, CRP and percentage of neutrophil count), of the 92 patients positive for appendicitis (Groups B and C), 81 patients had all the three values raised and only 11 patients had one or more values in the normal range. Among Group A, only one patient had all the three values raised and 17 patients had one or more values in the normal range [Table 1].

**Table 1:** Grouping of the patients as per the histology of the removed appendix

Group	Operative finding	No. of patients	TLC raised	CRP raised	Percentage of neutrophil count raised	TLC and CRP raised	All normal	All raised
A	Uninflamed appendix	18	8	4	11	2	14	1
B	Inflamed but uncomplicated appendix	79	77	76	78	66	Nil	69
C	Complicated appendix	13	13	12	13	11	Nil	12

TLC - Total leucocyte count, CRP - C-reactive protein

The sensitivity and specificity of TLC in this study were 97.82% and 55.55% and that for CRP were 95.6% and 77.77%. The positive predictive values for TLC and CRP were 91.8% and 95.6%, respectively (***P*** < 0.001). The combined TLC and CRP had a sensitivity, specificity and positive predictive value of 83.69%, 88.88% and 97.46%, respectively. When all the three parameters (TLC, CRP and percentage of neutrophil count) were combined, the specificity was increased to 94.44% and the positive predictive value improved to 98.7% [Table 2].

**Table 2:** Sensitivity, specificity and positive predictive value of TLC, CRP and percentage of neutrophil count

Parameter	Sensitivity	Specificity	Positive predictive value
TLC	97.82	55.55	91.8
CRP	95.6	77.77	95.6
Combined TLC and CRP	83.69	88.88	97.46
Combined TLC, CRP and percentage of neutrophil count	88.04	94.44	98.7

TLC - Total leucocyte count, CRP - C-reactive protein

Of the 18 cases negative for appendicitis, seven had clear-cut other diagnosis. The remaining patients had a final diagnosis of nonspecific abdominal pain [Table 3].

**Table 3:** Diagnosis other than appendicitis in the selected patients

Other diagnosis	No. of patients
Complicated ovarian cyst	4
Mesenteric lymphadenitis	1
Duodenal ulcer perforation	1
Meckel's diverticulitis	1

## DISCUSSION

Acute appendicitis is the most common cause of urgent abdominal surgery.[5] Because clinical diagnosis of acute appendicitis is difficult, appendectomy after false-positive diagnosis of appendicitis (hereafter, negative appendectomy) is performed in up to15–25% of the cases.[6] Some authors have even reported negative appendectomy rates of up to 50% in women of the reproductive age group.[7] Such negative explorations have been accepted as an unavoidable consequence of the principle of early exploration to prevent perforation of the appendix, but this practice is being questioned increasingly.

A majority of the patients with acute appendicitis present with right-sided lower abdominal pain, nausea and vomiting, but these symptoms are very nonspecific. In fact, any acute abdominal condition can mimic appendicitis and thus the list of differential diagnosis is long and hence removal of a normal appendix is not unusual. Localized tenderness and evidence of peritoneal inflammation (guarding and percussion tenderness) make the diagnosis probable. Laboratory investigations usually contribute little and can be misleading.[8]

Although appendicectomy is considered to be a safe operation, it has still got associated complications, most noticeable among them being wound infection, intraabdominal abscess, adhesions and bowel obstruction and pulmonary complications from general anesthesia. Additionally, some patients have persistent symptoms even after the surgery. Such patients constitute as a burden on the hospital resources while being generally unsatisfied with the health care providers.

Appendicectomy for a normal appendix is associated with both mortality and morbidity.[1] Some reports indicate a higher risk for intestinal obstruction following surgery for a normal appendix compared with that for a nonperforated inflamed appendix.[9,10] The risk for intestinal obstruction is increased by up to 5% in patients with a healthy appendix.[11]

CRP was identified in 1930 by Tillet and Francis and is regarded as the acute-phase protein.[12] It has been studied as a screening device for inflammation, a marker for disease activity and as a diagnostic adjunct. Physiologically, CRP enhances cell-mediated immunity by promoting phagocytosis, accelerating chemotaxis and activating platelets. CRP is a reliable early indicator of inflammation or injury.[12,13] Mustard ***et al.*** documented that serial postoperative CRP levels could predict septic complications before their clinical manifestation.[14]

Several studies have addressed the accuracy of CRP in diagnosing appendicitis and it is agreed that its level increases in appendicitis, which is related to the severity of appendiceal inflammation.[15,16] The CRP concentration is thus a very useful nonspecific biochemical marker of inflammation, measurement of which contributes importantly to (1) screening for organic disease, (2) monitoring of the response to treatment of inflammation and infection and (3) detection of intercurrent infection in immunocompromised individuals and in the few specific diseases characterized by modest or absent acute-phase responses.[17]

Although CRP increases with inflammation, it increases markedly after the occurrence of complications.[18] The increase in the leucocyte count is an early sign of appendix inflammation. CRP measurements or leucocyte counts alone are not effective in preventing negative appendectomies.[19]

Scores of studies have been conducted to determine the role of TLC and CRP in the diagnosis of acute appendicitis, with all giving varying results. The aim of our study was to determine the diagnostic accuracy of TLC, CRP and neutrophil count in combination in the diagnosis of acute appendicitis. Our study concluded that based on unaided clinical signs and symptoms, diagnostic accuracy of acute appendicitis was less than 80%. The sensitivity and specificity of TLC, CRP and percentage of neutrophil in the diagnosis of acute appendicitis was calculated individually and in combination. It was observed that when combined, the specificity and positive predictive value were raised, with a greatly improved probability of diagnosing acute appendicitis in equivocal cases.

## CONCLUSION

We concluded that if patients with right iliac fossa pain were explored on the basis of preoperative serum CRP levels and TLC counts, and due respect was given to the percentage of neutrophil count, eight out of 18 negative explorations would have been prevented thus preventing the morbidity and burden on hospital resources associated with these negative explorations. Therefore, we recommend performing all three of these laboratory tests in combination in patients with an equivocal diagnosis of acute appendicitis based on clinical signs alone, before surgical exploration.
